# *Circoviridae* Survey in Captive Non-Human Primates, Italy

**DOI:** 10.3390/ani14060881

**Published:** 2024-03-13

**Authors:** Vittorio Sarchese, Federica Di Profio, Andrea Palombieri, Klaus Gunther Friedrich, Serena Robetto, Krisztian Banyai, Fulvio Marsilio, Vito Martella, Barbara Di Martino

**Affiliations:** 1 Department of Veterinary Medicine, Università degli Studi di Teramo, 64100 Teramo, Italy; vsarchese@unite.it (V.S.); fdiprofio@unite.it (F.D.P.); apalombieri@unite.it (A.P.); fmarsilio@unite.it (F.M.); 2 Fondazione Bioparco, Viale del Giardino Zoologico, 00197 Rome, Italy; aorta333@yahoo.it; 3 Istituto Zooprofilattico Sperimentale del Piemonte, della Liguria e della Valle d’Aosta, S.S. Patologie della Fauna Selvatica, 11020 Quart, Italy; serena.robetto@izsto.it; 4 Institute for Veterinary Medical Research, Centre for Agricultural Research, 1143 Budapest, Hungary; bkrota@hotmail.com; 5 Department of Pharmacology and Toxicology, University of Veterinary Medicine, 1078 Budapest, Hungary; 6 Department of Veterinary Medicine, Università Aldo Moro di Bari, 70010 Valenzano, Italy; vito.martella@uniba.it

**Keywords:** *Circoviridae*, cyclovirus, non-human primates

## Abstract

**Simple Summary:**

Information on the host range and genetic diversity of members of the *Circoviridae* family is quickly increasing, but the ecology of these viruses remains largely unknown. In this study, using a panviral PCR targeting the Rep gene, we detected CyV DNA in rectal and saliva swabs collected from 48 NHPs housed in Bioparco—Rome Zoological Garden (Italy) and in the Anima Natura Wild Sanctuary Semproniano (Grosseto, Italy), with an overall prevalence of 18.7% (9/48). When reconstructing the sequence and genome organization of five strains, all CyVs appeared genetically highly related (98.3–98.6% nucleotide identity) to a CyV strain (RI196/ITA) detected in the intestinal content of a Maltese wall lizard (*Podarcis filfolensis*) in Italy.

**Abstract:**

Circoviruses (CVs) and cycloviruses (CyVs), members of the family *Circoviridae*, have been identified only occasionally in non-human primates (NHPs). In this study, we investigated the presence and genetic features of these viruses in 48 NHPs housed in the Bioparco—Rome Zoological Garden (Italy) and in the Anima Natura Wild Sanctuary Semproniano (Grosseto, Italy), testing fecal, saliva, and serum samples with a broadly reactive consensus nested PCR able of amplifying a partial region of the replicase (Rep) gene of members of the family *Circoviridae*. Viral DNA was detected in a total of 10 samples, including a saliva swab and 9 fecal samples collected, respectively from five Japanese macaques (*Macaca fuscata*) and four mandrills (*Mandrillus sphinx*), with an overall prevalence of 18.7% (9/48). On genome sequencing, five strains revealed the highest nucleotide identity (98.3–98.6%) to a CyV strain (RI196/ITA) detected in the intestinal content of a Maltese wall lizard (*Podarcis filfolensis*) in Italy. Although the origin of the Italian NHP strains, genetically distant from previously detected NHP CyVs, is uncertain, our results also highlight that the virome of captive animals is modulated by the different dietary and environmental sources of exposure.

## 1. Introduction

The family *Circoviridae* encompasses viruses with covalently closed, circular, single-stranded DNA (ssDNA) genomes of 1.7–2.1 kb length enclosed in non-enveloped, icosahedral virions [1]. Members of the family are classified into two genera, *Circovirus* and *Cyclovirus*. Circoviruses (CVs) and cycloviruses (CyVs) have an ambisense genome organization containing two major inversely arranged open reading frames (ORFs), encoding the replication-associated (Rep) protein and capsid (Cp) protein that are transcribed bidirectionally [2]. The stem-loop structure at the 5′ intergenic region containing the conserved sequence NANTATTAC has a role in the initiation of rolling-circle replication (RCR) [1]. *Circoviridae* replicate their genomes using a circular, double-strand (ds) replicative form (RF) DNA intermediate, which is produced using host cell DNA polymerases of actively dividing cells during the S phase. The RF serves as a template for generation of viral ssDNA using the RCR mechanism [1,2]. 

Over the last decade, with the development of high-throughput sequencing methods, an increasing number of CVs and CyVs has been identified from a diverse range of host animal species, including mammals [3,4], birds [5,6], freshwater fishes [7,8], and insects [9,10]. Based on genome-wide pairwise nucleotide [nt] sequence identities (with a species demarcation threshold of 80%) [1,2], the International Committee on Taxonomy of Viruses (ICTV), at least 60 species have been recognized within the genus *Circovirus* and 89 species within the genus *Cyclovirus* (https://ictv.global/taxonomy, accessed on 28 January 2024). 

Some mammalian and avian CVs are considered of high veterinary importance, i.e., PCV2-associated diseases in pigs (PCVADs) [11,12] and the beak and feather disease virus (PBFDV) of birds [13,14]. Infection in these animals is typically associated with potentially fatal illnesses, including multisystem wasting, hemorrhagic enteritis, vasculitis, and necrosis of lymphatic tissues [15,16]. Recently, two novel human CVs have been detected in a French patient with acute hepatitis of unknown origin [17], and in two Chinese drug users co-infected with human immunodeficiency virus 1 and hepatitis C virus [18]. Although the source of infection for these patients remained undetermined, a zoonotic pathway, potentially enacted by the compromised immune system, has been hypothesized [17,18].

CyVs were first discovered during a large molecular screening performed in Pakistan, Tunisia, and Nigeria on stool samples from humans, chimpanzees, and meat products of local cows, goats, sheep, camels, and chicken [19]. In total, 17 *Cyclovirus* species were identified in 395 human fecal specimens, and an additional 5 species were detected in muscular tissues from 204 farm animals. Interestingly, a single CyV species was found in common in both groups of samples. Since then, a variety of novel CyV species have been recovered from human samples other than feces, including cerebrospinal fluid specimens and serum samples collected from children and adults with central nervous system (CNS) infections of unknown origin [20] or with unexplained paraplegia [21]. CyV DNA has also been identified in blood samples from patients with symptoms resembling dengue [22] and in nasopharyngeal aspirates of children with acute lower respiratory tract infections [23]. 

Occasionally, CVs and CyVs have been detected in non-human primates (NHPs). Avian CV-like sequences similar to those identified in fecal specimens from Nigerian children have been found in 3 (6.8%) out of 44 fecal samples collected from wild chimpanzees (*Pan troglodytes*) in Central Africa [19]. Furthermore, four novel *Cyclovirus* species, genetically unrelated to other CyV strains identified in humans and farm animals, were detected in 14.0% (6/44) of the samples assessed [19]. More recently, taking advantage of high-throughput sequencing technologies and the metagenomic approach for virus discovery, novel CyVs have been identified in the stools of a drill monkey (*Mandrillus leucophaeus*) housed in a sanctuary in Cross River State (Nigeria) [24] and in salivary swabs from chimpanzees housed in two sanctuaries in the Republic of Congo and Uganda [25]. Overall, based on the available literature, genetically diverse circoviruses, for which cross-species transmission seems plausible, were identified in two different NHP species [19,24,25]. However, the relevance of the detection of these viruses in the NHPs population in terms of epidemiology, animal conservation, and zoonotic potential remains unclear. In the frame of the active monitoring and health assessments of captive NHP populations, circoviruses surveillance could be pivotal to investigate host range and clinical relevance not only for animal conservation but also for the assessment of zoonotic risks from a One Health perspective, also complementing studies performed in wild-living monkeys. In this study, we investigated the presence of *Circoviridae* in a collection of fecal, salivary, and serum samples obtained from captive NHPs of eight different species.

## 2. Materials and Methods

### 2.1. Sampling

Molecular screening was performed on paired serum (n = 24), salivary (n = 24), and fecal samples (n = 24) collected between July 2021 and June 2022 from a total of 24 NHPs, including 9 Japanese macaques (*Macaca fuscata*), 4 mandrills (*Mandrillus sphinx*), 3 ring-tailed lemurs (*Lemur catta*), 3 black lemurs (*Eulemur macaco*), 3 white-crowned mangabeys (*Cercocebus lunulatus*), and 2 red ruffed lemurs (*Varecia rubra*) (collection A), housed in the Bioparco—Rome Zoological Garden. In addition, 24 fecal swabs obtained from 11 common marmosets (*Callithrix jacchus*), 7 cynomolgus macaques (*Macaca fascicularis*), and 6 Japanese macaques housed in the Anima Natura Wild Sanctuary Semproniano (Grosseto, Italy) were collected in January 2023 and included in the screening (collection B). In both facilities, all the animals are housed in groups of NHPs belonging to the same species or individually. The animals of each NHP species are not allowed to have contact with any other captive animal species or to access outdoor environments. Finally, the holding areas were fenced by glass or narrow mesh nets. 

All the sampled animals were clinically healthy at the time of sampling. Specimens were collected during routine health examinations performed by veterinarians for the care and welfare of the NHPs and none of the animals were specifically bled for this study. Samples were stored at −70 °C in sterile containers before shipment to the Department of Veterinary Medicine, University of Teramo (Italy), where they were kept at −80 °C until testing.

### 2.2. Nucleic Acids Extraction and Molecular Investigation 

Each fecal sample (~100 μg) was homogenized in sterile phosphate-buffered saline (0.15 M, pH 7.2) solution to obtain a total volume of 1 mL and then centrifuged at 10,000× *g* for 3 min to collect the supernatant, whilst the saliva and serum samples were not diluted. Nucleic acids were extracted from 200 μL of each specimen using the Quick-DNA/RNA Viral MagBead Kit (Zymo Research, Irvine, CA, USA) according to the manufacturer’s instructions. Each nucleic acids extract was subsequently treated with the OneStep PCR Inhibitor Removal Kit (Zymo Research, Irvine, CA, USA) to remove potential PCR inhibitors. All samples were screened in triplicate by a nested PCR assay using broadly reactive primers (Pan-Rep) (Table 1) for members of the *Circoviridae* family and targeting, respectively in the first- and second-round PCRs, ~500 and ~400 nucleotide (nt) long fragments of the Rep gene [19]. First- and second-round PCR protocols were performed using GoTaq^®^ G2 Hot Start Taq Polymerase (Promega Italia S.r.l, Milan, Italy). Each reaction contained 12.5 μL of GoTaq^®^ G2 Hot Start Green Master Mix, 2X, 0.25 μL of each primer at a final concentration of 0.25 μM, 1 μL of the DNA template, and nuclease-free water to a final volume of 25 μL. The cycling conditions consisted of activation of the Hot-Start polymerase at 94 °C × 2 min, followed by 35 cycles of denaturation at 94 °C for 15 s, annealing at 52 °C for 15 s, and extension at 68 °C for 15 s. One microliter of the PCR product, diluted 1:100, was used as a template in the second-round PCR performed with the same protocol as the first-round PCR. The amplification products were run on a 1% agarose gel containing a fluorescent nucleic acid marker (GelRed^®^ Nucleic Acid Gel Stain; Biotium, Fremont, CA, USA) and visualized under ultraviolet (UV) illumination. Contamination was prevented through a severe separation between pre-amplification, amplification, and post-amplification procedures. PCR mixtures were prepared in a separate room. Microtubes containing DNA extracts, which were never taken into the PCR set-up room, were added to the PCR mixtures in separate laminar flow cabinets. Beyond workspaces separation, distilled water was used as negative control in each PCR run. All of the amplicons yielding bands of expected size were excised from gel, purified with the QIAquick gel extraction kit (Qiagen GmbH, Hilden, Germany), and subjected to Sanger sequencing from both directions using BigDye Terminator Cycle chemistry. The sequence chromatograms were generated on 3730 DNA Analyzer (Applied Biosystems, Foster, CA, USA). 

### 2.3. Genome Amplification and Sequence Analysis

Attempts to generate the complete genome were made on all the Pan-Rep PCR-positive samples. In detail, the viral DNA was enriched using a multiply primed rolling circle amplification (RCA), with the bacteriophage phi29 DNA polymerase (TempliPhi 100 amplification kit, Cytiva, Marlborough, MA, USA). Briefly, 5 µL of extracted DNA was added to a mix containing 4 µL of TempliPhi sample buffer and 1 µL of pan-Rep reverse primer CV-R1 [19]. After incubation at 95 °C for 3 min, 5 µL TempliPhi reaction buffer supplemented with 0.7 µL of 10 mM dNTPs (Invitrogen, Ltd., Paisley, Scotland, UK) and 0.2 µL TempliPhi enzyme were added to the annealing mix. The mixture was then incubated at 35 °C for 22 h and subsequently inactivated at 65 °C for 15 min [27,28]. Recovery of the viral circular genome was performed using an inverse (back-to-back) PCR strategy employing the primer sets 162R/201F and 117R/305F [18] and two additional internal primers 535R and 896F designed in this study and targeting the Cap gene sequence (Table 1). The inverse PCRs were performed with TaKaRa La Taq high-fidelity polymerase (TaKaRa Bio Europe S.A.S. Saint-Germain-en-Laye, France). The thermal conditions of the first-round PCR included an initial step at 94 °C × 2 min, followed by 35 cycles of 94 °C × 30 s, 60 °C × 30 s, and 68 °C × 3 min, with a final extension of 68 °C × 10 min. One microliter of a 1:100 dilution of the first-round PCR product was used in the second-round amplifications, using nested primers (117R/307F and 535R/896F). The thermal protocol was the same as for the first-round PCR. The amplicons generated in the inverse PCR were directly sequenced. The alignment of the sequences was performed using the MUSCLE multiple alignment program [29] version 3.8.425 plugin of the Geneious Prime Version 2022.2.2 (Biomatters Ltd., Auckland, New Zealand). The phylogenetic reconstruction was conducted using the Maximum Likelihood method, supplying statistical support with bootstrapping of 1000 replicates in MEGA 11 software [30].

## 3. Results

Using Pan-Rep primers, viral DNA was detected in a total of 9 NHPs with an overall prevalence of 18.7% (9/48). Amplicons of the expected size were found in five fecal samples collected, respectively from one Japanese macaque (11.1%, 1/9) and from the four mandrills (100%, 4/4) in collection A. Viral DNA was also detected in a salivary swab from a mandrill (25.0%, 1/4) that was also positive for the fecal sample, whilst all the serum samples tested negative. An additional four samples (66.7%, 4/6) obtained from Japanese macaques were found to contain viral DNA when testing the fecal specimens of collection B (Table 2). 

Partial Rep sequences (~400 nt) were determined from all the positive samples and compared with cognate sequences available in the databases. Upon sequence alignment and analysis of pairwise percent identities, all the sequences shared 99.1–100% nt identity to each other, whilst the overall nt identities to other CyVs ranged from 29.9% to 99.1%. The closest match (98.0–99.1% nt) was to a CyV strain (RI196/ITA) identified in the intestinal content of a Maltese wall lizard (*Podarcis filfolensis*) in Italy [26] and to human CyVs (69.7–72.4% nt) detected in nasopharyngeal aspirates from Chilean children less than 2 years old with acute lower respiratory infections [23].

The complete genome was generated for four CyVs of enteric origin, detected, respectively in a mandrill (strain NHP21F/2022/ITA, GenBank accession no. OR271549) and in three Japanese macaques (NHP39/2023/ITA, NHP40/2023/ITA and NHP41/ITA/2023, OR271551-OR271553) of the collection B, and for the strain detected in the saliva swab (NHP21S/2022/ITA, OR271550). Based on genome sequence alignment, the five Italian NHP CyVs displayed 99.3–100% pairwise genome-wide percent identity to each other, with the highest match found for the sequences identified in stool (NHP21F/2022/ITA) and saliva (NHP21S/2022/ITA) samples collected from the same mandrill of the collection A. The five genomes were each 1792 nt in length, with the two ORFs of 837 nt and 708 nt, coding, respectively for the putative Rep (278 aa) and Cp (235 aa) proteins. The termination of the Rep encoding gene overlapped the end of the Cp encoding gene by 8 nt. Similar to other CyVs, a putative *ori* of 255 nt in length, marked by a conserved nonanucleotide motif (TAATACTAT), was mapped at the apex of the predicted stem-loop structure [1,2]. The Rep protein of the five Italian NHP strains contained the three highly conserved Walker motifs of the SF3 helicase domain, including Walker A (^165^GPPGTGKS^174^), Walker B (^206^IIDDF^212^), and motif C (^246^ITSN^251^) [31] and the three rolling-circle replication motifs, including motif I (^9^FTLHDY^16^), motif II (^46^PHLQG^52^), and motif III (^86^YCSK^91^) [32,33]. 

**Figure 1:**
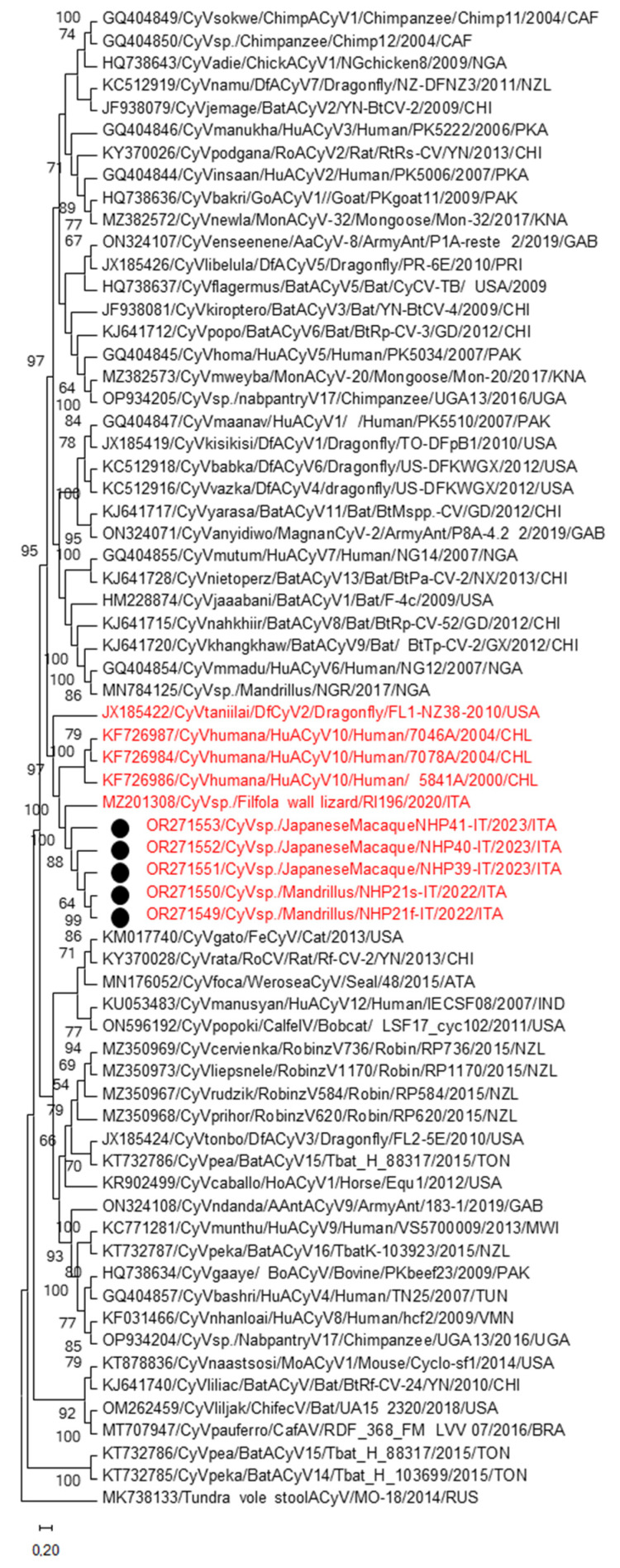
The evolutionary history was inferred by using the Maximum Likelihood method and Kimura 2-parameter model. The percentages of replicate trees in which the associated taxa clustered together in the bootstrap test (1000 replicates) are shown next to the branches. Evolutionary analyses were conducted in MEGA 11 [30]. The black circle indicates full-genome sequences generated in this study.

Upon interrogation (January 2024) of Genbank database, the strains NHP21F/2022/ITA, NHP21S/2022/ITA, NHP39/2023/ITA, NHP40/2023/ITA and NHP41/ITA/2023 displayed the best match (98.3–98.6% pairwise genome-wide nt identity) to the lizard CyV RI196/ITA [26], with pairwise aminoacid [aa] identities of 99.6–100% and 95.8–96.6%, respectively, in the full rep and Cp proteins. The full genomes of the five NHP strains were aligned with CyV complete genomic nucleotide sequences currently available on GenBank databases using MUSCLE [29] and the alignments were used to construct the phylogenetic tree. Maximum Likelihood tree (Figure 1) revealed that the NHPs CyVs grouped tightly (bootstrap value 100%) with the Italian strain RI196/ITA of lizard origin [26]. These strains displayed 98.3–100% nt identity to each other and represented the cluster genetically most close (71.2–71.7% nt identity) to members of the species *Cyclovirus umana* (7078A, 5841A, 7046A) detected in respiratory samples from Chilean patients [23]. The simian CyVs were also distantly related (47.4–47.6% nt identity) to the CyV strain FL1-NZ38-2010, which was identified in a dragonfly and classified in the species *Cyclovirus taniilai* [34]. The overall identity to other CyV complete genomes, including those previously detected in chimpanzees and mandrills [19,24,25], was <47.4%.

## 4. Discussion

Identification of viruses belonging to the family *Circoviridae* in NHPs has been documented [19,24,25]. In this study, we applied a Pan-Rep nested PCR protocol [19] to screen 96 samples, including serum (n = 24), salivary (n = 24) and fecal (n = 48) specimens collected from 48 NHPs housed in two different settings in Lazio (collection A) and Toscana (collection B). Using this approach, we identified Rep CyV-like sequences in 18.7% (9/48) of the NHPs tested, with detection rates of 20.8% (5/24) and 4.2% (1/24), respectively, in fecal and salivary specimens of collection A and of 16.7% (4/24) in fecal samples of collection B. These findings demonstrate the circulation of similar CyVs in NHPs of different settings, raising a number of questions, i.e., if these viruses have a pathogenic role in NHPs or they are innocuous components of simian virome.

In our study, all CyV-positive samples were collected from apparently healthy NHPs, indicating a lack of association between these viruses and clinical conditions. The virus was detected only in fecal samples and in a unique salivary sample, whilst none of the serum samples tested positive for CyV DNA. The presence of viruses in fecal samples can also be related to a dietary or environmental origin of the virus, which is passively transported through the gastrointestinal tract with infected/contaminated food, rather than being the result of active virus replication in the host. On the other hand, we observed that among the eight NHP species investigated, CyV DNA was detected only in Japanese macaques (33.3%, 5/15) and mandrills (100%, 4/4), whilst the other NHP species were negative, a finding that may be compatible with mechanisms of host species restriction or with different exposure linked to environment/dietary/behavior conditions.

Partial CyV Rep sequences were obtained from all the positive samples. According to sequence analyses, the ten partial sequences shared high identity (97.6–100% nt) and displayed the closest identity (98.6–99.1%) to a lizard strain, RI196/ITA, identified in 2022 in the Sicily region [26]. When reconstructing the complete sequence and genome organization of five NHP CyVs, the closest sequence identity (98.3–98.6%) to the lizard strain was confirmed. The origin of the NHP CyVs identified in this study is uncertain. However, the finding that the Italian NHPs CyVs appeared virtually identical to the strain RI196/ITA is intriguing, as RI196/Rep, along with other CyV Rep-like sequences, were recently identified at high detection rate (21.2%; 22/104) in fecal samples collected from synanthropic squamates (geckos and lizards) in different Italian areas [26]. Accordingly, we cannot exclude the possible role of squamates as environmental sources of exposure for NHPs assessed in this study. In addition, considering that CyVs are frequently detected in arthropods [34], it could be also possible that the identification of similar virus in lizards and NHPs was due to being exposed to or eating the same CyVs invertebrate hosts, which are ubiquitous in Italian territories. Indeed, in both structures housing the NHPs investigated in this study, animals were kept in areas fenced by narrow mesh net that could not be guaranteed to avoid the entry of arthropods and small mammals, amphibians, or reptiles and, notably, it is recognized that NHPs could prey and eat small animals as part of curiosity-driven, exploratory behaviors, but also as part of food-seeking behavior [35]. In addition, as lizards are considered common synanthropic animals and the NHPs investigated were captive animals which interacted more easily with the veterinary and caretaker employees, the possible role of humans as potential viral sources could be taken into consideration, since several CyVs have been identified from different human samples and conditions [19,20,21,22,23]. Interestingly, genetically, the NHP CyVs viruses were related (71.2–71.7% pairwise genome-wide nt identity) either in the Rep (pairwise genome aa identity of 78.1–78.4%) or Cap (pairwise genome aa identity of 55.9–56.3%) proteins to human CyV strains 7078A, 5841A, and 7046A of respiratory origin [23]. Strictly following the ICTV classification criteria for species demarcation (threshold of 80%) of *Circoviridae* family [1,2], the NHPs CyVs detected in this study should be classified together with the strain RI196/ITA [18] as members of a candidate novel species within the genus *Cyclovirus*, which is sister to the species *Cyclovirus umana*. However, considering the large gap in percent identity, it could be hypothesized that in the wide diversity of CyVs, the actual closest related viruses to the NHP and lizard CyVs have not been yet identified or described.

## 5. Conclusions

Our study extended the available information in terms of the geographic distribution, host range, and also genetic diversity of CyVs. Additional studies are warranted to elucidate the relevance of these findings and to establish the epidemiological and clinical importance of CyVs in NHPs. The identification of animal-like CVs in humans [17,18] and of CyVs in patients with CNS infections of unknown origin [20,21,36] has fueled the research on these viruses, unveiling that CyVs have broad host range and a low species-specificity. Exploring the virome of animals is important in terms of animal conservation and fulfills the requirements of the One Health paradigm. Contextually, the findings of our study also highlight that NHPs living in captivity and inhabiting geographic areas at great distances from their ecoregions experience substantially different environmental conditions compared to their wild counterparts in terms of diet, climatic conditions, movement space, and close proximity to humans, that could drive the captive NHP viral exposure, shaping their virome.

## Figures and Tables

**Table 1:** List of oligonucleotides used in this study.

Assay	Primers	Sequence (5’-3’)	Amplification Size (nt)	Reference
Pan-Rep PCR	CV-F1	GGIAYICCICAYYTICARGG	500	[19]
	CV-R1	AWCCAICCRTARAARTCRTC		
nestedPCR	CV-F2	GGIAYICCICAYYTICARGGITT	400	
	CV-R2	TGYTGYTCRTAICCRTCCCACCA		
Inverse PCR	162R	CTGGCTCAATCTAACACATCCTAT	>1500	[26]
	201F	GTGTAACTCGGCAATACGTTTAAT		
nestedPCR	117R	TGAAGTATCATACTACTGGGGGC		
	305F	TTTCAAAGTATTCGCCCGATTTAG		
nestedPCR	535R	TGAGTTCCTCTGTATACTTTAGA	351	This study
	896F	TATAGAAGGCGTTTTATGAGAT		

**Table 2:** NHP species tested in this study and assessed with Pan-CV nested PCR.

	NHP Species Tested	No. of Animals	Type of Samples Total % (Positive/Total)			Animals Total % (Positive/Total)
			**Serum**	**Saliva**	**Faeces**	
**Collection A**	Japanese macaque	9	0% (0/9)	0% (0/9)	11.1% (1/9)	11.1% (1/9)
	Mandrill	4	0% (0/4)	25% (1/4)	100% (4/4)	100.0% (4/4)
	Ring-tailed lemur	3	0% (0/3)	0% (0/3)	0% (0/3)	0.0% (0/3)
	Black lemur	3	0% (0/3)	0% (0/3)	0% (0/3)	0.0% (0/3)
	White-crowned mangabey	3	0% (0/3)	0% (0/3)	0% (0/3)	0.0% (0/3)
	Red ruffed lemur	2	0% (0/2)	0% (0/2)	0% (0/2)	0.0% (0/2)
**Collection B**	Common marmoset	11	n.a.	n.a.	0.0% (0/11)	0.0% (0/11)
	Cynomolgus macaque	7	n.a.	n.a.	0.0% (0/7)	0.0% (0/7)
	Japanese macaque	6	n.a.	n.a.	66.7% (4/6)	66.7% (4/6)
**Total**		**48**	**0% (0/24)**	**4.2% (1/24)**	**18.7% (9/48)**	**18.7% (9/48)**

n.a. = not available.

## Data Availability

The data supporting the findings of this study are openly available in the GenBank database with accession number OR271549-OR271553 and OR271606-OR271610.

## References

[1] Rosario K., Breitbart M., Harrach B., Segalés J., Delwart E., Biagini P., Varsani A. (2017). Revisiting the taxonomy of the family Circoviridae: Establishment of the genus Cyclovirus and removal of the genus Gyrovirus. Arch. Virol..

[2] Breitbart M., Delwart E., Rosario K., Segalés J., Varsani A., Ictv Report Consortium (2017). ICTV Virus Taxonomy Profile: Circoviridae. J. Gen. Virol..

[3] Li L., Shan T., Soji O.B., Alam M.M., Kunz T.H., Zaidi S.Z., Delwart E. (2011). Possible cross-species transmission of circoviruses and cycloviruses among farm animals. J. Gen. Virol..

[4] Lian H., Liu Y., Li N., Wang Y., Zhang S., Hu R. (2014). Novel circovirus from mink, China. Emerg. Infect. Dis..

[5] Todd D. (2014). Circoviruses: Immunosuppressive threats to avian species: A review. Avian Pathol..

[6] Johne R., Fernandez-de-Luco D., Hofle U., Muller H. (2006). Genome of a novel circovirus of starlings, amplified by multiply primed rolling-circle amplification. J. Gen. Virol..

[7] Doszpoly A., Tarján Z.L., Glávits R., Müller T., Benkő M. (2014). Full genome sequence of a novel circo-like virus detected in an adult European eel Anguilla anguilla showing signs of cauliflower disease. Dis. Aquat. Organ..

[8] Lőrincz M., Dán A., Láng M., Csaba G., Tóth A.G., Székely C., Cságola A., Tuboly T. (2012). Novel circovirus in European catfish (*Silurus glanis*). Arch. Virol..

[9] Padilla-Rodriguez M., Rosario K., Breitbart M. (2013). Novel cyclovirus discovered in the Florida woods cockroach Eurycotis floridana (Walker). Arch Virol..

[10] Rosario K., Mettel K.A., Benner B.E., Johnson R., Scott C., Yusseff-Vanegas S.Z., Baker C.C.M., Cassill D.L., Storer C., Varsani A. (2018). Virus discovery in all three major lineages of terrestrial arthropods highlights the diversity of single-stranded DNA viruses associated with invertebrates. PeerJ.

[11] Segalés J. (2012). Porcine circovirus type 2 (PCV2) infections: Clinical signs, pathology and laboratory diagnosis. Virus Res..

[12] Segalés J., Kekarainen T., Cortey M. (2013). The natural history of porcine circovirus type 2: From an inoffensive virus to a devastating swine disease?. Vet. Microbiol..

[13] Julian L., Piasecki T., Chrząstek K., Walters M., Muhire B., Harkins G.W., Martin D.P., Varsani A. (2013). Extensive recombination detected among beak and feather disease virus isolates from breeding facilities in Poland. J. Gen. Virol..

[14] Massaro M., Ortiz-Catedral L., Julian L., Galbraith J.A., Kurenbach B., Kearvell J., Kemp J., van Hal J., Elkington S., Taylor G. (2012). Molecular characterisation of beak and feather disease virus (BFDV) in New Zealand and its implications for managing an infectious disease. Arch. Virol..

[15] Baekbo P., Kristensen C.S., Larsen L.E. (2012). Porcine circovirus diseases: A review of PMWS: Porcine circovirus diseases. Transbound. Emerg. Dis..

[16] Fogell D.J., Martin R.O., Groombridge J.J. (2016). Beak and feather disease virus in wild and captive parrots: An analysis of geographic and taxonomic distribution and methodological trends. Arch. Virol..

[17] Pérot P., Fourgeaud J., Rouzaud C., Regnault B., Da Rocha N., Fontaine H., Le Pavec J., Dolidon S., Garzaro M., Chretien D. (2023). Circovirus hepatitis infection in heart-lung transplant patient, France. Emerg. Infect. Dis..

[18] Li Y., Zhang P., Ye M., Tian R.R., Li N., Cao L., Ma Y., Liu F.L., Zheng Y.T., Zhang C. (2023). Novel Circovirus in Blood from Intravenous Drug Users, Yunnan, China. Emerg. Infect. Dis..

[19] Li L., Kapoor A., Slikas B., Bamidele O.S., Wang C., Shaukat S., Masroor M.A., Wilson M.L., Ndjango J.B.N., Peeters M. (2010). Multiple diverse circoviruses infect farm animals and are commonly found in human and chimpanzee feces. J. Virol..

[20] Tan L.V., van Doorn H.R., Nghia H.D.T., Chau T.T.H., Tu L.T.P., de Vries M., Canuti M., Deijs M., Jebbink M.F., Baker S. (2013). Identification of a new Cyclovirus in cerebrospinal fluid of patients with acute central nervous system infections. mBio.

[21] Smits S.L., Zijlstra E.E., van Hellemond J.J., Schapendonk C.M.E., Bodewes R., Schürch A.C., Haagmans B.L., Osterhaus A.D.M.E. (2013). Novel Cyclovirus in human cerebrospinal fluid, Malawi, 2010–2011. Emerg Infect Dis..

[22] Yozwiak N.L., Skewes-Cox P., Stenglein M.D., Balmaseda A., Harris E., DeRisi J.L. (2012). Virus identification in unknown tropical febrile illness cases using deep sequencing. PLoS Negl. Trop. Dis..

[23] Phan T.G., Luchsinger V., Avendano L.F., Deng X., Delwart E. (2014). Cyclovirus in nasopharyngeal aspirates of Chilean children with respiratory infections. J. Gen. Virol..

[24] George U., Simsek C., Faleye T.O.C., Arowolo O., Oragwa A., Adewumi O.M., Matthijnssens J., Adeniji J.A. (2020). Genome Sequences of Novel Members of Previously Described DNA and RNA Virus Families, Isolated from Feces of a Drill Monkey in Nigeria. Microbiol. Resour. Announc..

[25] Dunay E., Rukundo J., Atencia R., Cole M.F., Cantwell A., Emery Thompson M., Rosati A.G., Goldberg T.L. (2023). Viruses in saliva from sanctuary chimpanzees (Pan troglodytes) in Republic of Congo and Uganda. PLoS ONE.

[26] Capozza P., Lanave G., Diakoudi G., Pellegrini F., Cardone R., Vasinioti V.I., Decaro N., Elia G., Catella C., Alberti A. (2022). Diversity of CRESS DNA Viruses in Squamates Recapitulates Hosts Dietary and Environmental Sources of Exposure. Microbiol. Spectr..

[27] Wu L., Liu X., Schadt C.W., Zhou J. (2006). Microarray-based analysis of Subnanogram quantities of microbial community DNAs by using whole-community genome amplification. Appl. Environ. Microbiol..

[28] Johne R., Müller H., Rector A., van Ranst M., Stevens H. (2009). Rolling-circle amplification of viral DNA genomes using phi29 polymerase. Trends Microbiol..

[29] Edgar R.C. (2004). MUSCLE: Multiple sequence alignment with high accuracy and high throughput. Nucleic Acids Res..

[30] Tamura K., Stecher G., Kumar S. (2021). MEGA11: Molecular Evolutionary Genetics Analysis Version 11. Mol. Biol. Evol..

[31] Walker J.E., Saraste M., Runswick M.J., Gay N.J. (1982). Distantly related sequences in the alpha- and beta-subunits of ATP synthase, myosin, kinases and other ATP-requiring enzymes and a common nucleotide binding fold. EMBO J..

[32] Rosario K., Duffy S., Breitbart M. (2012). A field guide to eukaryotic circular single-stranded DNA viruses: Insights gained from metagenomics. Arch. Virol..

[33] Dayaram A., Potter K.A., Moline A.B., Rosenstein D.D., Marinov M., Thomas J.E., Breitbart M., Rosario K., Argüello-Astorga G.R., Varsani A. (2013). High global diversity of cycloviruses amongst dragonflies. J. Gen. Virol..

[34] Rosario K., Dayaram A., Marinov M., Ware J., Kraberger S., Stainton D., Breitbart M., Varsani A. (2012). Diverse circular ssDNA viruses discovered in dragonflies (Odonata: Epiprocta). J. Gen. Virol..

[35] Watts D.P. (2020). Meat eating by nonhuman primates: A review and synthesis. J. Hum. Evol..

[36] Cyclovirus in Cerebrospinal Fluid of Patients with Central Nervous System Infection. https://www.ecdc.europa.eu/sites/default/files/media/en/publications/Publications/rapid-risk-assessment-Cyclovirus-final.pdf.

